# Strength gain through eccentric isotonic training without changes in clinical signs or blood markers

**DOI:** 10.1186/1471-2474-14-328

**Published:** 2013-11-21

**Authors:** Thâmara Alves, Flávia A Guarnier, Fernanda AS Campoy, Mariana O Gois, Maíra C Albuquerque, Patrícia M Seraphim, Jayme Junior Netto, Luiz Carlos Marques Vanderlei, Carlos R Padovani, Rubens Cecchini, Carlos Marcelo Pastre

**Affiliations:** 1 Department of General Pathology, State University of Londrina, Londrina, Brazil; 2 Department of Physical Therapy, Univ Estadual Paulista, Presidente Prudente, Brazil; 3 Department of Physical Therapy, Federal University of São Carlos, São Carlos, Brazil; 4 Department of Biostatistics, Univ Estadual Paulista, Botucatu, Brazil

**Keywords:** Training programs, Muscle strength, Pain, Blood markers

## Abstract

**Background:**

Localized exercises are widely used in rehabilitation processes. The predominant options are exercises with an emphasis on either concentric or eccentric contractions. Eccentric exercises promote greater strength gains compared to classical concentric stimuli, but can cause muscle damage. The aim of present study was to compare strength training composed of 10 sessions with progressive loads between groups with a predominance of concentric *versus* eccentric contraction through an analysis of isotonic strength, pressure pain threshold, creatine kinase, tumor necrosis factor-alpha and cortisol.

**Methods:**

One hundred twenty male subjects were divided into four groups: C1 and E1 – single session of maximum strength with emphasis on concentric and eccentric contraction, respectively; C10 and E10 – 10 sessions with progressive loads from 80% to maximum strength with emphasis on concentric and eccentric contraction, respectively.

**Results:**

Isotonic strength increased by 10% in E10 following the ten training sessions. C1 and E1 exhibited a lower pressure pain threshold 48 hours after the sessions in comparison to C10 and E10, respectively. Creatine kinase was increased in C1 in comparison to baseline, with significant differences (p ≤ 0.05) in comparison to E1 at 48 and 96 hours as well as C10 at 48, 72 and 96 hours. No significant differences were found in TNF-α or cortisol among the groups or evaluation times.

**Conclusion:**

Eccentric contraction training promotes functional adaptation. Moreover, both concentric and eccentric contraction training have a protective effect on the muscle in relation to a single session of maximum strength exercise.

**Trial registration:**

RBR-75scwh

## Background

Localized exercise is widely used in rehabilitation processes. Diverse techniques administered in clinical practice place an emphasis on concentric and eccentric contractions, with the aim of enhancing muscle strength [1,2]. However, studies comparing these two forms of contraction indicate differences in the responses [2-4]. A number of studies state that eccentric contraction has advantages over concentric contraction [4-6], such as the greater synthesis of myofibrillar proteins and hypertrophy [3], greater torque and lesser energy expenditure [4]. However, eccentric contractions are reported to induce greater muscle damage, which can lead to clinical and functional deficits, as represented by an increase in serum levels of muscle-specific proteins, pro-inflammatory cytokines and blood markers of physiological stress [7-9].

These observations are found in studies carried out to investigate functional/clinical responses and blood markers after exercise sessions predominantly involving eccentric contractions performed with loads ranging from 80 to 150% of one-repetition maximum (1RM) and considerable variation with regard to the work volume [7-11]. Moreover, a review reveals no studies that jointly analyze both the adaptive processes of training on clinical and functional variables and the response to exertion. Therefore, obtaining positive and negative outcomes after performing exercise at different paces in untrained subjects can add elements of interest to the literature.

The aim of the present study was to compare strength training (10 sessions) with different characteristics (rapid concentric and slow eccentric phase *versus* slow concentric and rapid eccentric phase) and a single session of maximum exercise on the knee extensor group through an analysis of isotonic strength (functional variable), the pressure pain threshold (clinical variable) and blood markers (creatine kinase, tumor necrosis factor-alpha and cortisol).

## Methods

### Study population

One hundred twenty male subjects between 18 and 26 years old and classified as physically active by the International Physical Activity Questionnaire [12] participated in this study. The following were the exclusion criteria: anemia, diabetes, cardiovascular disease, self-reported liver damage, alcoholism, drug use, tobacco use, chronic use of anti-inflammatory medication, episode of muscle/tendon or osteoarticular injury in the lower limbs or back in the previous year, participation in muscle-building program in the previous six months and known inflammatory or infectious process in the previous week [13].

The subjects were informed with regard to the objectives and procedures of the study and agreed to participate by signing a statement of informed consent. This statement also contained a declaration that the subjects had seen a physician who ensured them of adequate physical fitness to perform the exercises. Confidentiality was ensured and the study received approval from the Human Research Ethics Committee of the School of Sciences and Technology, *UNESP - Univ Estadual Paulista* (Brazil) under protocol number 20/2010.

### Experimental design

**Figure 1:**
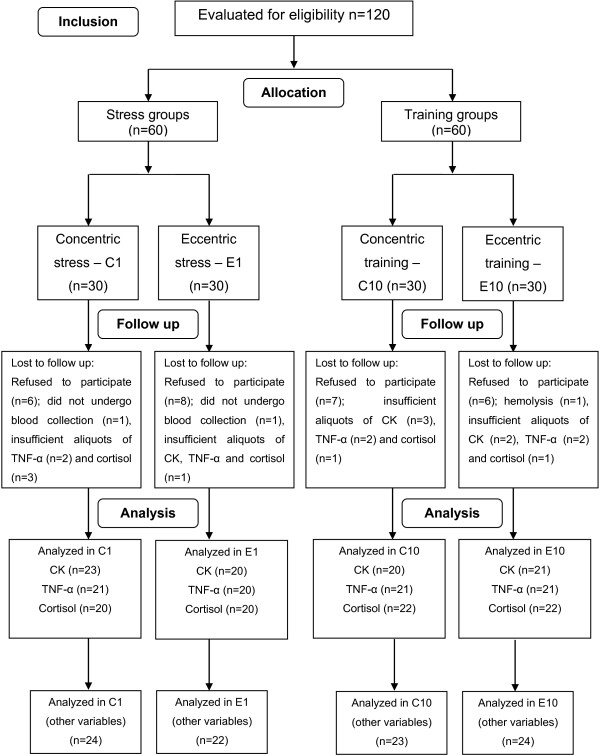
**Distribution of the volunteers in each group and losses throughout the study.** Legend - CK: creatine kinase; TNF-α: tumor necrosis factor alpha.

Prior to the procedures, data were collected on the name, age and anthropometric data [body mass, height and body mass index (BMI)] of each subject. The volunteers were randomly divided into four groups based on the protocol (maximum exercise or training) and type of contraction emphasized: concentric stress group (C1), eccentric stress group (E1), concentric training group (C10) and eccentric training group (E10). Figure 1 displays the flowchart of the distribution of the volunteers in each group and losses throughout the study.

Prior to the data collection, one week was dedicated exclusively to the 1RM test and familiarization of the participants with the equipment and exercise protocol. The 1RM test indicated the maximum load (in kilograms) that each individual was able to lift during knee extension, which was subsequently used to determine the training loads. Table 1 displays the evolution of the training loads, number of repetitions and rest interval between sets.

**Table 1:** Training according to exercise dynamics and sessions

**Sessions**	**Work volume**	**Intensity**	**Interval between sets**
**1 and 2**	3 sets of 8 repetitions	80% of 1RM	3 minutes
**3 and 4**	3 sets of 6 repetitions	85% of 1RM	2 minutes
**5 and 6**	3 sets of 4 repetitions	90% of 1RM	1 minute 30 seconds
**7 and 8**	3 sets of 2 repetitions	95% of 1RM	40 seconds
**9 and 10**	3 sets of 1 repetitions	100% of 1RM	20 seconds

Legend - 1RM: One-repetition maximum.

One week after the 1RM test and familiarization with the protocol, blood was collected and information was gathered on the pressure pain threshold (PPT) of the rectus femoris muscle. C10 and E10 then underwent ten strength training sessions at a frequency of three times a week on alternate days for three weeks. C1 and E1 were submitted to a single exercise session corresponding to the last training session performed in C10 and E10 (3 sets of one repetition at 100% 1RM). Table 2 displays the evaluation times for each variable in C10 and E10. These times were chosen based on the acute and chronic responses to training to gain a better understanding of the behavior of the variables analyzed. In C1 and E1, the collection of the variables coincided with the evaluations times used for C10 and E10.

**Table 2:** Distribution of evaluation times according to variables

	**T0**	**T1**	**T2**	**T3**	**T4**
**1RM**	X				X
**PPT**	X	X	X	X	X
**CK**	X	X	X	X	X
**TNF-α**	X	X	X	X	X
**Cortisol**	X	X			

Legend - 1RM: One-repetition maximum; PPT: Pressure pain threshold; CK: Creatine kinase; TNF-α: Tumor necrosis factor-alpha. T0: baseline; T1: 24 hours after 10^th^ session; T2: 48 hours after 10^th^ session; T3: 72 hours after 10^th^ session; T4: 96 hours after 10^th^ session.

The exercise dynamics were based on the classic progressive load method, respecting the interdependence of volume and intensity proposed by Chiesa [14] and adapted to the progress needs in the ten sessions. Thus, the protocol emphasized the gain in muscle strength recommended in clinical practice and physical therapy, ensuring the specificity of the work and individuality in the load progression. The sessions were always held between 6 and 8 pm to minimize physiological variations and alterations stemming from the circadian rhythm, as suggested by Miles et al. [13] and Teo et al. [15].

### Exercise protocols

C1 and C10 performed knee extension in three seconds beginning with 90° flexion until 180° extension to emphasize concentric contraction, with the return of the limb to the original position occurring within a one-second period (slow concentric and fast eccentric contraction). E1 and E10 performed knee extension in one second beginning with 90° flexion until 180° extension with the return of the limb to the original position occurring within a three-second period to emphasize eccentric contraction (fast concentric and slow eccentric contraction). The three-second periods were determined based on Corvino et al. [16], who state that the strength development rate is dependent on the velocity of the contraction, with greater strength obtained when using slow movements.

The exercises were performed with the non-dominant limb. According to Glesson et al. [17], the dominant limb may be influenced by concentric and eccentric physical effort during activities of daily living, thereby potentiating the effect of the gain in muscle strength.

### Isotonic strength

Isotonic strength was measured based on the 1RM test, which was begun at 50% of the subject’s body weight, with increases of 30% and based on the subject’s perception until reaching the maximum load with which the subject could execute the movement without mechanical failure. No more than five attempts were permitted for the determination of maximum load; otherwise the test was considered invalid and the subject had to repeat the procedure another day [18].

A muscle building device (*Ipiranga, Academia Hard line*) was used for the execution of the exercise, during which the subject remained seated with hands supported on the sides of the chair to promote greater stability during the movement. Velcro™ straps were attached to the trunk and thighs to minimize compensatory movements.

### Pressure pain threshold

The pressure pain threshold of the rectus femoris muscle was determined using a pressure algometer (*Wagner Instruments, FPX 50/220*), which was validated in a study by Kinser et al. [19]. The PPT was expressed in Kgf based on the minimum pressure necessary to induce discomfort in the thigh. The device was positioned over the rectus femoris at a site corresponding to 30% of the length of the thigh (measured between the upper anterior iliac spine to the upper edge of the patella). Pressure was applied slowly and evenly and, for reasons of safety, did not exceed 2.55 Kgf, as suggested by Jönhagen et al. [20].

### Blood samples

Five-mL blood samples were taken from the antecubital vein in heparinized tubes for the subsequent analysis of creatine kinase (CK), tumor necrosis factor-alpha (TNF-α) and cortisol on plasma samples. Total blood tubes were kept at 4°C until centrifugation, which was performed at 1500 rpm for 10 minutes at 4°C. The plasma was obtained and stored at -20°C until the analyses.

### Creatine kinase

The investigation of possible muscle damage caused by the eccentric *versus* concentric exercises was performed by the evaluation of total CK concentration in the blood sample using the *CK-NAC* and *CKmb-NAC* colorimetric kits (*Laborclin*) for readings in a spectrophotometer (*SP-220, Biospectro*) at 340 nm. Differences in absorbance were recorded and the final results of both enzymes were expressed as U/L.

### Tumor necrosis factor-alpha and cortisol

The determination of TNF-α and cortisol concentrations was performed using enzyme-linked immunosorbent assays commercial kits (*eBioscience®* for TNF-α, and *IBL America®* for cortisol). At the end of procedures, final absorbance was read at 450 nm in a universal microplate reader (*Biotek, Biosystems*), as recommended by the manufacturers. The results were expressed as pg of TNF-α/mL or ng of cortisol/mL.

### Data analysis

The sample size was calculated based on isotonic strength as the central variable, with a standard deviation of 7 Kg (determined in a pilot study) and an estimated difference of 6 Kg between baseline and 96 hours after stimulus. A two-tailed hypothesis test was performed, with a 5% level of significance and 80% test power. It was determined that a minimum of 21 subjects per group were needed.

The groups were initially compared to identify the homogeneity of the sample regarding the anthropometric variables and age. The Kolmogorov-Smirnov test was used to test the normality of the data distribution. Parametric two-way repeated-measures analysis of variance (ANOVA) was used to compare groups, types of contractions and evaluation times regarding isotonic strength, PPT, TNF-α and cortisol and non-parametric ANOVA was used for CK, complemented by the Student-Newman-Keuls test and Dunn’s test, respectively. The level of significance was set to 5% (p ≤ 0.05) [21].

## Results

No significant differences were found between groups regarding anthropometric variables or age (Table 3).

**Table 3:** Mean and standard deviation values of anthropometric variables and age of subjects in stress and training groups

	**Groups**			
**Variables**	**C1**	**E1**	**C10**	**E10**
	**N=24**	**n=22**	**n=23**	**n=24**
Age (years)	22.17±2.94	21.41±2.54	20.56±2.83	21.33±3.23
	21.50	21.00	20.00	20.00
Body mass (Kg)	70.37±11.20	75.27±9.95	71.18±12.11	73.95±11.29
	68.50	75.50	70.00	74.50
Height (m)	1.73±0.05	1.76±0.09	1.74±0.07	1.75±0.07
	1.73	1.75	1.73	1.74
BMI (kg/m^2^)	23.58±3.86	24.21±2.48	23.58±3.13	24.05±3.58
	22.46	24.53	23.46	24.26

Legend: C1: Concentric stress; E1: Eccentric stress; C10: Concentric training; E10: eccentric training.

The eccentric training group (E10) exhibited a significant increase in isotonic strength at the 96-hour evaluation (p ≤ 0.05) in comparison to the baseline evaluation as well as in comparison to E1 (E10: 49.58 ± 11.32 at baseline and 55.42 ± 10.52 at 96 hours; E1: 47.96 ± 13.94 at baseline and 46.36 ± 14.90 at 96 hours). C1 and E1 had a lower PPT (Kgf) in comparison to C10 and E10, respectively, 48 hours after the session (C1: 2.26 ± 0.48; E1: 2.36 ± 0.34; C10: 2.48 ± 0.19; E10: 2.53 ± 0.09). C1 exhibited an increase in CK at 24 (68.75), 48 (111.30), 72 (90.02) and 96 (91.90) hours in comparison to baseline (54.58) and differed significantly (p ≤ 0.05) from E1 at 48 (65.04) and 96 (39.84) hours as well as C10 at 48 (29.77), 72 (29.82) and 96 (27.00) hours (Table 4).

**Table 4:** Mean and standard deviation of 1RM test and PPT and median of CK concentration according to group, type of contraction and evaluation time

**Variables**	**Groups**	**Evaluation time**				
		**Baseline**	**24 hours**	**48 hours**	**72 hours**	**96 hours**
**1RM (Kg)**	**C1** (n=24)	46.67±15.72				46.04±16.02
	**E1** (n=22)	47.96±13.94				46.36±14.90
	**C10** (n=23)	46.30±10.03				48.70±11.20
	**E10** (n=24)	49.58±11.32				55.42±10.52 * #
**PPT (Kgf)**	**C1** (n=24)	2.34±0.36	2.37±0.34	2.26±0.48	2.34±0.39	2.31±0.41
	**E1** (n=22)	2.48±0.22	2.47±0.22	2.36±0.34	2.36±0.36	2.49±0.13
	**C10** (n=23)	2.46±0.29	2.45±0.29	2.48±0.19 *	2.48±0.26	2.50±0.23
	**E10** (n=24)	2.54±0.04	2.52±0.11	2.53±0.09 #	2.50±0.18	2.46±0.31
**CK (U/L)**	**C1** (n=24)	54.58	68.75 ♦	111.30 * # ♦	90.02 * ♦	91.90 * # ♦
	**E1** (n=22)	44.17	66.34	65.04	55.76	39.84
	**C10** (n=23)	25.57	26.98	29.77	29.82	27.00
	**E10** (n=24)	31.50	34.05	37.13	39.64	34.87

Legend: C1: Concentric stress; E1: Eccentric stress; C10: Concentric training; E10: Eccentric training; 1RM: One-repetition maximum – * p < 0.05 in comparison to baseline; # p < 0.05 in comparison to G2 at 96 hours; PPT: Pressure pain threshold – * p < 0.05 in comparison to C1; # p < 0.05 in comparison to E1; CK: Creatine kinase – * p < 0.05 in comparison to C10; # p < 0.05 in comparison to E1; ♦ p < 0.05 in comparison to baseline.

No significant differences were found in TNF-α or cortisol among the groups or evaluation times (Table 5).

**Table 5:** Mean and standard deviation values for TNF-α and cortisol concentrations according to group, type of contraction and evaluation time

**Variables**	**Groups**	**Evaluation time**				
		**Baseline**	**24 hours**	**48 hours**	**72 hours**	**96 hours**
**TNF-α (pg/mL)**	**C1** (n=24)	11.16±0.08	11.17±0.08	11.16±0.07	11.17±0.10	11.17±0.08
	**E1** (n=22)	11.17±0.12	11.15±0.07	11.16±0.09	11.15±0.07	11.15±0.07
	**C10** (n=23)	11.18±0.07	11.18±0.08	11.17±0.10	11.19±0.10	11.20±0.11
	**E10** (n=24)	11.20±0.07	11.20±0.09	11.18±0.06	11.19±0.07	11.20±0.09
**Cortisol (ng/mL)**	**C1** (n=24)	96.32±17.30				93.48±16.83
	**E1** (n=22)	90.97±17.95				93.95 ±16.56
	**C10** (n=23)	91.19±19.59				89.23±12.61
	**E10** (n=24)	94.95±16.40				91.24±16.31

Legend: C1: Concentric stress; E1: Eccentric stress; C10: Concentric training; E10: Eccentric training. TNF-α: Tumor necrosis factor alpha.

## Discussion

In the present study, 10 sessions of eccentric contraction training led to a significant increase in isotonic strength in comparison to a single session of eccentric stress. Moreover, both stress groups exhibited a decrease in PPT and the concentric stress group exhibited an increase in CK levels between 24 and 96 hours after the session.

The group trained with an emphasis on the slow eccentric contraction phase achieved an approximately 10% increase in isotonic strength, which was a significant difference in comparison to mean baseline strength. This is in agreement with findings reported in the literature that a greater gain in strength is achieved with eccentric training [3-6]. A number of authors report that sessions of eccentric exercise are more effective at stimulating the synthesis of myofibrillar proteins and collagen in comparison to concentric contraction of equal volume and intensity [3-6]. According to Moore et al. [3], maximum eccentric contractions produce greater strength than concentric contractions of the same intensity, even though eccentric contraction leads to the recruitment of a lesser amount of muscle fibers.

Besides the increase in isotonic strength in E10 at the end of the training period, a significant difference was found in the results of the 1RM test between E1 and E10 after the sessions, indicating adaptation in the group that underwent training in comparison to the group submitted to a single session of maximum eccentric exercise.

Both stress groups exhibited a reduction in the PPT 48 hours following the session at the site corresponding to 30% of the length of the thigh in comparison to the groups that underwent concentric and eccentric training. This finding also demonstrates adaptation in the training groups, regardless of the type of contraction employed. Andersen et al. [22] found central adaptations of pain perception after resistance training. The authors demonstrated that PPT of trained painful trapezius and the non-trained reference muscle of the leg increased more in the training groups compared with the control group. These data are in agreement with findings reported in the present study that demonstrated adaptation in the training groups in comparison to the stress groups.

As demonstrated above, gains of strength require cell adaptation [3,10], which is a phenomenon that requires a cell challenge. There are no positive effects without some kind of cell injury or disturbance and a network of signaling molecules are responsible for gains in functional parameters, including molecules involved in injurious and inflammatory processes [23]. It seems that the most efficient protocol is one that is capable of promoting functional gain with less tissue damage. In the present study, a significant increase in CK was found in the concentric stress group. In contrast, no changes in TNF-α or cortisol concentrations were found either within or between groups, indicating the absence of an inflammatory process or an alteration in the pattern of physiological stress.

Besides being a good indicator of cell damage, CK also demonstrates disturbances regarding cell permeability [10]. The analysis of the CK demonstrated a significant increase in the level of this enzyme in the concentric stress group 24, 48, 72 and 96 hours after the session. Moreover, the concentric stress group had higher CK levels in comparison to the eccentric stress and concentric training groups at 48 and 96 hours. These results differ from findings described in the literature reporting higher CK levels following eccentric exercise in comparison to concentric exercise [4,7]. However, there may be an interpretation bias when declaring that eccentric exercise causes more damage. The load in the present study went up to the maximum limit of the volunteers, whereas the load in the studies citied surpassed this limit. Thus, the present findings suggest that eccentric exercise is not more aggressive than concentric exercise when respecting the maximum limit of the individual.

Regarding the fact that maximum concentric stress led to a significant increase in CK, a large portion of the subjects in the concentric stress and training groups exhibited greater difficulty performing the exercise protocol in comparison to those in the eccentric stress and training groups. This difficulty may have resulted in an increase in the intensity of the effort during the maximum stress session due to the slow concentric contraction, leading to the increase in CK levels in C1. However, the median values in this group did not surpass the reference value of the kit used for males (38 to 174 U/L) and were therefore within the acceptable range of serum levels for this enzyme. Nonetheless, a greater tendency toward an increase in CK was found in C1, as 21.7% of the participants had values higher than the reference value 48 hours following the session.

The concentric stress group had higher CK levels in comparison to the concentric training group at 48, 72 and 96 hours. The subjects in the concentric training group likely underwent an adaptation process that resulted in unaltered CK levels at the end of the training period. The literature reports that the amount of muscle damage following a single session of localized maximum exercise is lessened in subsequent sessions due to the adaptation of the muscle fibers following the first session, resulting in a less accentuated increase or even the absence of an increase in serum CK following subsequent stimuli [7,8,10]. Indeed, Vissing et al*.*[7] and Chen et al*.*[8] found that the concentration of CK increased considerably following one session of eccentric exercise and adaptation occurred in subsequent sessions, resulting in a reduction in serum levels of this enzyme. The authors report that the reduction in muscle damage is possibly due to the replacement of less resistant fibers.

In the present study, the concentric stress group exhibited an increase in CK levels and a decrease in PPT 48 hours after the session, but without the presence of an inflammatory process, as evident by the unaltered concentrations of TNF-α. Excluding the possibility of inflammation in the muscle fibers, the release of CK may indicate an increase in membrane permeability. According to Peake et al. [23], this may be related to the activation of calcium degradation pathways. Peake et al. [24] also found an increase in the release of CK, pain and decrease in PPT following resistance exercise, but found no changes in inflammatory markers, which is in agreement with the present findings.

No significant differences were found in TNF-α or cortisol between groups or evaluation times following the stress and training sessions. The literature reports divergent results regarding the concentration of these substances following resistance exercises. As in the present study, Hirose et al*.*[25] found no increase in TNF-α concentration following maximum eccentric exercises. The authors state that localized exercises exhibit little or no variation in serum levels of pro-inflammatory cytokines, whereas endurance exercises pose a considerable metabolic demand and consequently a greater stimulus to the secretion of stress hormones, which contribute significantly to the production of pro-inflammatory cytokines. In contrast, Silva et al*.*[11] found a significant increase in the serum concentration of TNF-α 48 hours following sessions of maximum eccentric exercise and state that such exercises may stimulate the production of pro-inflammatory cytokines as a response to muscle damage.

The unaltered concentration of cortisol may have been due to the lack of significant muscle damage following the stress and training sessions. According to Peake and Coombes [26], cells of the immune system are mobilized and activated during physical exercise in response to muscle damage and the action of stress-related hormones. Willoughby et al. [27] found no change in serum levels of cortisol following maximum concentric and eccentric exercises of the quadriceps. In contrast, Goto et al. [28] report an increase in the concentration of this hormone following predominantly eccentric exercises involving this same muscle group.

The considerable differences in protocols and methodological aspects in the studies consulted hamper the comparison of the results. However, the work volume and intensity proposed in the present study for the training sessions were within a safe range that promoted an increase in isotonic strength in E10 without producing significant damage to skeletal muscle that would lead to an increase in serum levels of CK, TNF-α or cortisol. Moreover, the training sessions had a protective effect, as demonstrated by the absence of muscle damage following the training sessions in comparison to the stress sessions. This finding is explained by the adaptation phenomenon, in which changes caused by the first stimulus result in the attenuation or even absence of muscle damage in subsequent sessions [8].

Since there was no inflammation (represented by the unaltered TNF-α) and there was a significant reduction in the PPT, it is reasonable to suppose that the concentric stress group experienced a permeability disturbance that was reflected in the decreased of the PPT.

A limitation of the present study was the lack of an analysis of PPT beyond that found in the rectus femoris, as a large portion of the volunteers in the stress groups and some in the training groups reported discomfort in the knee region, especially in the patellar ligament.

Based on the present findings, a training program with increasing loads involving fast concentric and slow eccentric contractions is safe for healthy individuals. The use of this protocol in physical therapy clinical practice should be encouraged with the aim of gaining strength in a short period of time, as it promotes an increase in isotonic muscle strength following ten sessions of exercise without inducing muscle damage.

## Conclusions

In conclusion, strength training based on rapid concentric and slow eccentric contractions promotes functional adaptation with an increase in isotonic strength. Moreover, a series of training sessions with increasing loads until reaching the maximum load provides a protective effect for the musculature, regardless of the type of contraction emphasized. When no systematic training is employed, a single session of localized exercise at 100% maximum load with an emphasis on slow concentric contraction can induce greater muscle damage in comparison to exercise with an emphasis on slow eccentric contraction of the same intensity without the installation of the inflammatory process.

## Abbreviations

ANOVA: Analysis of variance; BMI: Body mass index; CK: Creatine kinase; C1: Concentric stress group; E1: Eccentric stress group; C10: Concentric training group; E10: Eccentric training group; TNF-α: Tumor necrosis factor-alpha; UNESP: *Univ Estadual Paulista*; 1RM: One-repetition maximum.

## Competing interests

The authors declare that they have no competing interests.

## Authors’ contributions

TA and CMP were the main authors. CMP conceived of the study and TA, FASC, MOG, MCA and LCMV participated in the development of its design. TA, FAG, RC and PMS analyzed the blood markers. CRP performed the statistical analysis. TA drafted the manuscript. CMP, JNJ, LCMV and FAG revised the manuscript critically for important intellectual content. All authors read and approved the final manuscript.

## Pre-publication history

The pre-publication history for this paper can be accessed here:

http://www.biomedcentral.com/1471-2474/14/328/prepub
